# Statistical optimization of photo-induced biofabrication of silver nanoparticles using the cell extract of *Oscillatoria limnetica*: insight on characterization and antioxidant potentiality

**DOI:** 10.1039/d0ra08206f

**Published:** 2020-12-15

**Authors:** Rasha A. Abo-Elmagd, Mervat H. Hussein, Ragaa A. Hamouda, Ahmed Esmail Shalan, Ahmed Abdelrazak

**Affiliations:** Botany Department, Faculty of Science, Mansoura University Mansoura Egypt ahmed_bt@mans.edu.eg; Department of Biology, Faculty of Sciences and Arts Khulais, University of Jeddah Jeddah Saudi Arabia ragaahom@yahoo.com; Department of Microbial Biotechnology, Genetic Engineering & Biotechnology Research Institute, Sadat University Sadat City Egypt; Central Metallurgical Research and Development Institute (CMRDI) P.O. Box 87, Helwan Cairo 11421 Egypt a.shalan133@gmail.com; BCMaterials, Basque Center for Materials, Applications and Nanostructures Martina Casiano, UPV/EHU Science Park, Barrio Sarriena s/n Leioa 48940 Spain

## Abstract

Silver nanoparticles were successfully fabricated through a very simple, rapid, one-step photo-induced green approach. The formation of silver nanoparticles was accomplished using the bioactive compounds in the aqueous extract of fresh *Oscillatoria limnetica* biomass, which acted as a reducing and capping agent at the same time. The biosynthesis of *Oscillatoria*-silver nanoparticles (O-AgNPs) was investigated under the influence of different light intensities 57.75, 75.90 and 1276.51 μmol m^−2^ s^−1^ (bright sunlight). UV-Vis (UV) and Fourier transform infrared (FT-IR) spectroscopy were applied to approve the synthesis of AgNPs. Further, the synthesis process under the exposure to sunlight was adjusted *via* utilizing one factor at a time, and 0.5 mM AgNO_3_ concentration, 5 mL *O. limnetica* solution, pH 6.7 and 30 min sunlight (1276.51 μmol m^−2^ s^−1^) were applied. Furthermore, the central composite design (CCD) was applied to boost the biosynthesis process of O-AgNPs (manufactured at light intensity 75.90 μmol m^−2^ s^−1^). The maximum production of O-AgNPs was attained with 4 detected variables: initial pH level (6.7), AgNO_3_ concentration (0.3 mM), *O. limnetica* extract concentration (3.50 mL) and incubation time (48 h). Moreover, TEM, in addition to SEM, images exposed that the biosynthesized AgNPs were quasi-spherical in shape with a small monodisperse nature, and the size range was between 6.98–23.48 nm in the case of light-induced synthesis (75.90 μmol m^−2^ s^−1^) and 11.58–22.31 nm with sunlight (1276.51 μmol m^−2^ s^−1^).

## Abstract 2

Silver nanoparticles were successfully fabricated through a very simple, rapid, one-step photo-induced green approach.

## Graphical abstract

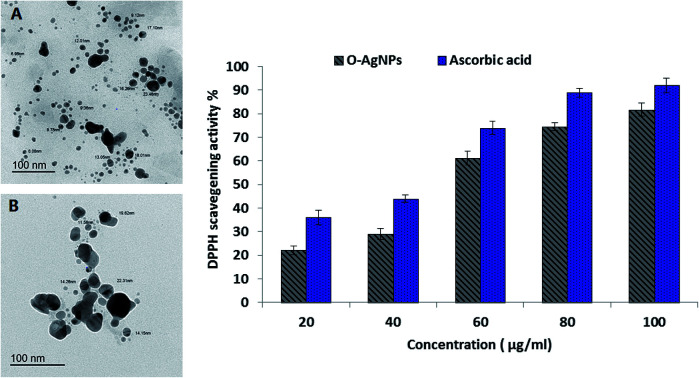

## Introduction

Nano-biotechnology is a promising and emerging domain among the modern key technologies and deals with the fabrication, design and manipulation of nanoscale materials with innovative applications using the asset of biological sources that affect the characteristics of the prepared nanoparticles (NPs). Recently, nano-biotechnology has opened novel frontiers in the development of diverse disciplines, including biomedical sciences, drug-gene delivery, cancer nanotechnology, chemical industries, catalysis, cosmetics, agriculture and wastewater treatment.^1^ The manufacture of metal nanoparticles (MNPs) have gained great attention due to their distinctive structural properties, small size (1–100 nm), large surface area, high reactivity and numerous applications in several advanced areas of science, including electronic devices, biomedicine, pharmaceuticals, biomarkers, biosensors and textile industries.^2^ Metal nanoparticles have characteristic chemical and physical features compared with their bulk materials as a consequence of their large surface-area-to-volume ratio.^3^ Furthermore, the broad usage of MNPs is expected to have a significant role in increasing the extension of the macroeconomic industry in the upcoming years.^4^ However, the escalating toxicity and rise in the price of MNPs may be the restricting factors that can eventually decrease their market value.^5^ Among the different MNPs, silver nanoparticles (AgNPs) are widely utilized because of their medical, as well as pharmaceutical, applications as strong antimicrobial, antioxidant, antibiofilm and anti-inflammatory agents and as catalysts for quickening some chemical reactions.^6^ Additionally, AgNPs can also be specifically applied in biosensing, gene therapy, wound dressings, medical/surgical tools and water treatment.^7^ Silver nanoparticles (AgNPs) have attained significant attention due to their efficient antimicrobial mediatory factors, which reveal low toxicity, besides their broad spectrum of *in vitro* and *in vivo* applications.^6^ Nowadays, several physical, chemical and biological methods are applied to fabricate nanoparticles. However, chemical and physical approaches for nanoparticle synthesis are widely used and effectual methodologies. Undesirably, they have some downsides, such as the use of high energy or toxic chemicals, work-intensified, high cost and the release of huge amounts of harmful by-products that cause great danger to living organisms and have an unfavorable influence on environmental and medical implementation.^8^ Consequently, there is an obvious basic necessity for an eco-friendly, low-cost, single-step, non-toxic and biocompatible approach for nanoparticle fabrication.^9^ Recently, the development of green routes that can remove or reduce the production of toxic chemicals and solvents and generate a sustainable system for the manufacture of metal nanoparticles has been highlighted as an alternate methodology.^10^ Thus, extracts from plants and algae,^11^ bacteria^12^ and fungi^13^ have become more attractive for environmentally benign biosynthesis of metallic nanoparticles. Numerous types of biomolecules in the extracts, such as enzymes, amino acids, proteins, carbohydrates, flavonoids, and terpenoids, which facilities in metal reduction and their stabilization, have been used in the biosynthesis of nanoparticles.^14^ Currently, phyconanotechnology has been an interesting and approaching domain with greater options in the production of algae-mediated nanoparticles. Algae are a source of a cluster of secondary metabolites, pigments and proteins that can function as nanobiofactories of metallic nanoparticles *via* extracellular or intracellular reduction of metal ions to their corresponding nano-forms.^15–19^ The abundance of algae and their ability for excessive metal uptake makes them an inexpensive raw material.^20^ The main purpose of this work was to achieve eco-friendly, and green photo-synthesis of stable silver nanoparticles (AgNPs) from the aqueous extract of *Oscillatoria limnetica* by photoinduction. The biosynthesis process was investigated using the central composite design (CCD) to get the optimum concentration of *O. limnetica*, pH, incubation period and concentration of AgNO_3_ for the preparation of AgNPs. Additionally, the characterization of the optimized biosynthesized AgNPs was executed by transmission electron microscopy (TEM), scanning electron microscopy (SEM), zeta potential calculation, as well as particle size analysis. Further, the antioxidant features of the attained AgNPs were estimated *via* the DPPH, ABTS, and erythrocyte assays.

## Materials and methods

### *Oscillatoria limnetica* culture environment and preparation of its extract

The cyanobacterium *Oscillatoria limnetica* was obtained from the algal culture collection of the phycology laboratory at Mansoura University. Besides, the axenic inoculum was cultured inside 500 mL Erlenmeyer flasks containing 200 mL of BG11 medium^21^ and incubated at 28 ± 1 °C under incessant illumination (57.75 μmol m^−2^ s^−1^) for almost 22 days at the start of the stationary growth stage. Furthermore, the synthesis of the extract was conducted according to ref. 22. For the preparation of the extract, fresh algal biomass was harvested from 200 mL of the culture by centrifugation at 1610 × *g* for 10 min using a centrifuge (Centurion Scientific, United Kingdom). Then, the obtained pellet corresponding to 0.154 g dry biomass per L was suspended in 10 mL deionized water and sonicated for 15 min at amplitude 100 using an ultrasonic homogenizer (Cole–Parmer instrumental Co. Chicago, Illinois 66648 USA). The homogenous suspension was made up to 100 mL with deionized water and stored at 4 °C for future use. In addition, the *O. limnetica* extract was chemically analysed for determining the content of light-harvesting pigments,^23^ proteins^24^ and total soluble sugars.^25^

### Light-mediated biosynthesis of *Oscillatoria*-silver nanoparticles

The silver source for AgNP production was silver nitrate powder (analytical grade AgNO_3_ with high purity). In this study, a 0.5 mM AgNO_3_ stock solution was prepared with distilled H_2_O and stored in a brown bottle at 4 °C for further use.

The cyanobacterial extract was used as the bio-mediator for the fabrication of AgNPs. The light-induced biosynthesis of *Oscillatoria*-silver nanoparticles (O-AgNPs) was conducted by following the protocol of Hamouda *et al.*^22^ The synthetic reaction was carried out by incubating the *O. limnetica* extract (5 mL of the prepared homogenous solution was made up with (0.1 M) phosphate buffer at pH 7 to 19 mL) with 1 mL AgNO_3_ solution (0.5 mM). The bio-reduction of O-AgNPs was accomplished using the following three light intensities: 57.75 (4273.5 lux), 75.90 (5616.6 lux) and 1276.51 (68931 lux) μmol m^−2^ s^−1^. The first two light intensities were achieved by incubating the mixtures at 35 ± 1 °C for 48 hours under the direct illumination of 36 W white fluorescent lamps, while 1276.51 μmol m^−2^ s^−1^ (68931 lux) light intensity was achieved with incubation under direct sunlight on a summer day (39 °C) at noon for 30 minutes. This condition was chosen after investigating the optimum period of sunlight exposure (5–30 min) and bio-reduction in the dark condition by keeping the mixtures in a closed container at the temperature and light intensity of 30 °C and 0 lux, respectively. The reaction mixtures revealed an instantaneous change in color from greenish to dark brown, signifying the synthesis of AgNPs. After the incubation time, the reduction of silver ions was assessed by measuring the absorption in the range of 200–900 nm using a UV-visible spectrophotometer (ATI Unicam 5625 UV/VIS Vision Software V3.20). Then, the reaction mixtures were centrifuged at 4528 × *g* for 10 min using a centrifuge (MIKRO 12 Hettich Zentrifugen D-78532 Tuttlingen-Germany) to separate the AgNPs. For complete purification, the precipitated Ag nanoparticles were washed 5 times with deionized water. Additionally, the obtained AgNPs were dried and stored for further examination.

### Optimization of O-AgNP biosynthesis using the central composite design (CCD)

The response surface methodology (RSM) was employed to explore potential interactions among the selected significant parameters that influence the production of O-AgNPs. The central composite design (CCD) proposed by McDonald *et al.*^26^ is a proficient statistical method that is applied for sequential experimentation to evaluate the optimization of variables and responses. The initial pH level (*X*_1_), AgNO_3_ concentration (mM) (*X*_2_), *O. limnetica* extract concentration (mL) (*X*_3_) and incubation period (h) (*X*_4_) are the main parameters that influence the *O. limnetica*-mediated synthesis of silver nanoparticles.^22^ They were used for optimizing the synthetic process *via* the central composite design (CCD). Furthermore, a matrix consisting of 31-trials was employed, and each factor was tested at five levels (coded, −2, −1, 0, +1, +2 and each trial was conducted in duplicate). The CCD matrix included seven center points and star points to assess the twist, which facilitates the prediction of noteworthy deficiency in the fitting of the statistical representations. The optical density (OD) that achieved the highest production of AgNPs at the wavelength of 430 nm was considered as a function in calculating the AgNP yield, which represents the reliance of the response (*Y*) on a variable. All the optimized silver nanoparticle samples were characterized *via* UV-Vis spectroscopy after each trail of AgNP synthesis, and the computed responses were the optical density values (OD_430_).

Additionally, the CCD experimental data were tailored through the response surface regression method *via* the next second-order polynomial equation.1
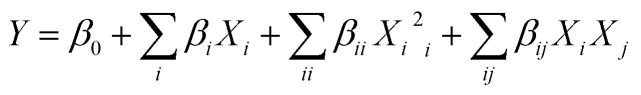
*Y* represents the predicted response, *β*_0_ represents the regression coefficients, *β*_*i*_ represents the linear coefficients, *β*_*ii*_ represents the quadratic coefficients, *β*_*ij*_ represents the interaction coefficients, and *X*_*i*_ represents the coded levels of the independent variables. Besides, the analysis of variances (ANOVA) was conducted *via* the Design Expert 8.0 statistical package (statEase, Inc, Minneapolis, MN, USA).

### Characterization of O-AgNPs

Confirmation of O-AgNP formation was attained by analyzing the absorption spectra of the biofabricated O-AgNPs *via* the UV-Vis spectroscopy technique (ATI Unicam 5625 UV/VIS Vision Software V3.20). Furthermore, to detect the biomolecules that lead to the reduction of Ag^+^ ions in addition to the capping of O-AgNPs, Fourier transforms infrared (FTIR) spectroscopy was used (ThermoFisherscientific Nicolete IS10, USA) in the window of 4000 to 500 cm^−1^ at a resolution of 1 cm^−1^, and the biofabricated AgNPs were lyophilized and diluted using KBr (1 : 100) to prepare a pellet. Besides, transmission electron microscopy (TEM) was applied to elucidate the accurate morphology, shape and particle size of the biosynthesized O-AgNPs (JEOL, JEM-2100, Japan). SEM analysis was utilized to investigate the surface characteristics (JEOL JSM-6510/v, Japan). In addition, the size, distribution and surface charge of the biogenic nanoparticles were determined using the zeta potential and particle size analyzer (Malvern Zeta sizer Nano-ZS90, UK).

### Biotechnological applications of O-AgNPs (antioxidant capacity of the photo-synthesized O-AgNPs)

The optimized photo-synthesized O-AgNPs (at the light intensity of 75.90 μmol m^−2^ s^−1^) were used for studying the antioxidant potentiality as follows:

#### DPPH free radical scavenging assay

1,1-diphenyl-2-picryl-hydrazyl (DPPH), a stable free radical compound, was applied to study the radical-scavenging activity of the produced O-AgNPs, according to Mani *et al.*^27^ O-AgNPs at the following concentrations: 20, 40, 60, 80 and 100 μg mL^−1^ were mixed with 1 mL of 0.1 mM DPPH solution and made up to 3 mL with methanol. Thereafter, the reaction mixtures were vigorously shaken and incubated at 37 °C for 30 min. The reduction of the DPPH free radical was quantified by measuring the absorbance at 517 nm using the ascorbic acid solution (0.01 mg mL^−1^) as the positive control. Besides, the free-radical-scavenging potential (reducing power) of O-AgNPs was evaluated *via* the below relationship:

2 DPPH scavenging effect (%) = 100 × (*A*_o_ − *A*_1_)/(*A*_o_)

where *A*_o_ represents the absorbance of the control reaction, and *A*_1_ represents the absorbance of the reaction mixture. The antioxidant activities of the nano-preparations are presented as IC_50_ values, which is the concentration of the extract that inhibits the formation of DPPH radicals by 50%, as indicated by the linear regression analysis.^28^

#### ABTS radical-scavenging assay

The ABTS radical-scavenging assay was conducted by following the protocol of Re *et al.*^29^ The bluish-green ABTS [2,2′-azino-bis(3-ethyl benzothiazoline-6-sulfonic acid)] radical was generated through the oxidation reaction resulting from mixing ABTS (7 mM) with potassium persulphate (2.45 mM). Then, the mixture was allowed to stand in the dark overnight for the formation of ABTS radicals (ABTS^+^) and diluted with distilled H_2_O to achieve an absorbance value of 1.00 at 734 nm, which was used for evaluating the antioxidant potentiality of the O-AgNP preparations. The reactivity of the ABTS cation radical enabled the investigation of antioxidants, including phenolic, thiols and ascorbic acids.^30^ The antioxidant activity is presented as the percent of inhibition and was calculated as follows using ascorbic acid (2 mM) as the positive control.3
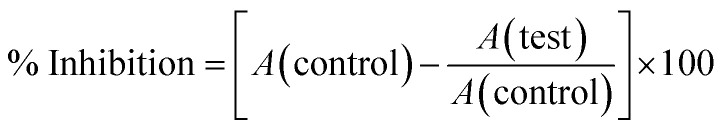


#### Antioxidant activity screening assay for erythrocyte hemolysis

Hemolysis is the consequence of a disorder in the integrity of the erythrocyte membrane, resulting in haemoglobin leakage into the blood plasma. The method applied for determining haemolysis was according to Morimoto *et al.*^31^

## Results and discussion

### Confirmation of O-AgNPs using spectral analysis

**Fig. 1:**
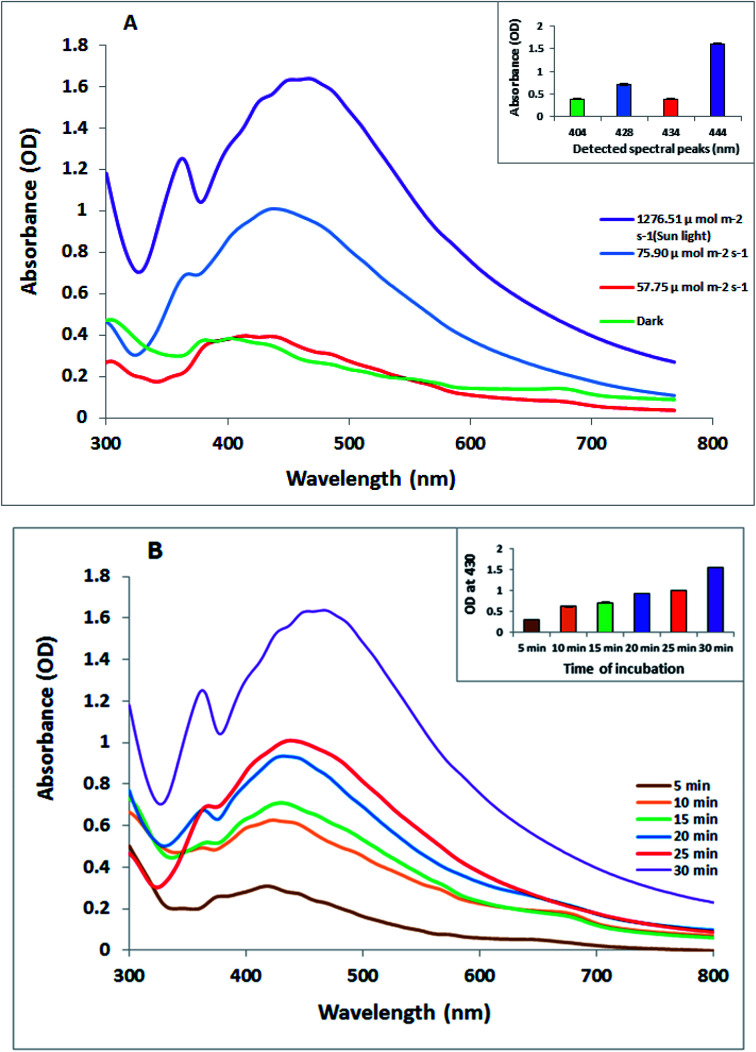
(A) UV-visible absorption spectra of the photo-synthesized *O. limnetica* silver nanoparticles (O-AgNPs) recorded as a function of different light intensities: 57.75, 75.90 and 1276.51 μmol m^−2^ s^−1^ (sunlight) and in the dark (conditions: *O. limnetica* extract solution 5 mL and 0.5 mM AgNO_3_ solution), and (B) the UV-visible absorption spectra of O-AgNPs recorded at different reaction times from 5 to 30 min under bright sunlight exposure (1276.51 μmol m^−2^ s^−1^).

The biosynthesis of silver nanoparticles was performed using the cyanobacterial extract of *O. limnetica*. The greenish aqueous *O. limnetica* extract was treated with 1 mL of an AgNO_3_ (0.5 mM) solution after incubation under a light. In this reaction, the color of the Ag^+^/*O. limnetica* reaction mixtures changed from the initial green to yellow and finally to brown as the reaction time increased, signifying bio-reduction and the formation of O-AgNPs of various particle sizes.^32^ The change in color was attributed to the excitation of the surface plasmon absorption of the formed O-AgNPs,^33^ as investigated by UV-Vis spectroscopy, since previous studies have indicated that the positioning of a strong and broad SPR peak between 400–500 nm confirms the formation of AgNPs.^34^ From the UV-Vis absorption spectrum, certain information about the size and shape of the synthesized AgNPs could be detected. The UV-Vis absorption spectrum of the photo-synthesized O-AgNPs from *O. limnetica* is shown in Fig. 1A. Additionally, the O-AgNPs revealed distinctive bands between 400 and 500 nm owing to surface plasmon resonance, and the broad plasmon peak that prolonged from 400 to 500 nm might be owing to the multi-size distribution of the photo-biofabricated AgNPs. As shown in Fig. 1A, absorption peaks were detected at the following wavelengths: 434, 428, 444 and 404 nm, giving the maximum optical intensities of 0.393, 0.709, 1.607 and 0.383 nm in the light regimes of 57.75, 75.90 and 1276.51 μmol m^−2^ s^−1^ (sunlight) and the dark, respectively, because of the formation of O-AgNPs. Interpretation of the current data led to the conclusion that the biosynthetic process of O-AgNPs using the *O. limnetica* extract in the dark might be dependent on proteins, soluble sugars, and polysaccharides, as well as reductases. The light-mediated biosynthesis of AgNPs demonstrated the necessity of the coordinated roles of light-harvesting pigments along with intracellular cell components, most certainly proteins and carbohydrates.^35^ These results could be attributed to the concentration and particle size of biosynthesized AgNPs.^36^ An approximate biochemical composition showing the light-harvesting pigment content in the *O. limnetica* aqueous extract is demonstrated in Table 1. Some postulations have previously suggested that the mechanism of the reduction of silver cations involves three characteristic electron-donating bio-catalysts, including protein/enzymes,^37^ polysaccharides^38^ and light-harvesting pigments.^39^ The interpretation is that AgNP biosynthesis might be achieved in two-steps: the silver cations would adhere to the algal cell surface in the aqueous medium due to the electrostatic attraction between the metal cations and the negatively charged cell wall, and then, metal cations would be reduced to the nano-form by the cellular reductases.^40^ Respecting to the presence of extracellular reductases such as nitrate reductases of *Scenedesmus obliquus* culture that using as reducing agents are supposed to have an essential role in the extracellular formation of AgNPs.^2^ In addition, the findings of Mukherjee *et al.* have documented their contribution towards the extracellular production of AgNPs using the *Bacillus clausii* culture.^41^ Studies have documented the role of protein molecules as both reducing and capping agents in the biogenic preparation of AgNPs besides their role toward stabilizing the AgNP preparations.^16,35,37,42^ Microbial EPS composed of proteins, polysaccharides, lipids, and some inorganic substances can function as a reducing and stabilizing agent in the biosynthesis of AgNPs.^43^ Light-induced procedures for the fabrication of nanoparticles are based on the reduction of the metal cation Mn^+^ to Mn^0^ by a direct or indirect pathway *via* photosystem II.^32^ Herein, the presence of *O. limnetica* phycobilisomes, which contain phycobiliproteins (colored protein-based pigments), and chlorophyll a-luxuriant thylakoids that assisted conformational alterations to absorb light energy for initiating photosynthesis. Excitation of the molecules from the ground state to an electronically excited state is mediated by chromospheres after absorbing light.^44^ Therefore, the proposed explanation is, under illumination, the electrons that jump between energy levels can as well reduce AgNO_3_ in the medium to produce silver nanoparticles.

**Table 1:** Phytochemical analysis of the *O. limnetica* extract

Total carbohydrates (mg g^−1^ dry wt)	Protein (mg/g day biomass)	Photosynthetic pigments (mg g^−1^)		Phycobiliproteins (mg mL^−1^)		
		Chlorophyll a (mg g^−1^)	Carotenoids (mg g^−1^)	Phycocyanin	Phycoerythrin	Allophycocyanin
306.667	225.455	18.06	2.27	0.313	0.087	0.169

Consequently, the optimal time for the sunlight-mediated biosynthesis of AgNPs (Fig. 1B) was obtained by monitoring the biofabrication of AgNPs under sunlight (5–30 min) at 5 min intervals, and the other experimental parameters were maintained at the same level (5 mL of *O. limnetica* aqueous solution and 1 mM silver nitrate solution). Besides, it was noticed that once the reaction mixture was exposed to bright sunlight, the physical appearance of the solution modified instantly, showing a rise in the intensity of color to deep brown with exposure time. We could notice that the absorbance was directly related to exposure time, as the absorbance value increased on increasing the exposure time, achieving its maximum value at 30 min. Interestingly, the peak maxima for exposure to diverse sunlight durations while the peak maxima was documented at 444 nm for 30 min of sunlight demonstrated that the size of O-AgNPs increased as the reaction time increased.^44^ The photo-mediated synthesis of O-AgNPs was initiated by the absorption of light by the photosensitive molecules present in the *O. limnetica* extract (Table 1).^45^ Consequently, 30 min under bright sunlight was measured as the ideal time for the photo-induced biofabrication of O-AgNPs and was used for additional characterization.

### FTIR spectroscopy of the photo-synthesized O-AgNPs

**Fig. 2:**
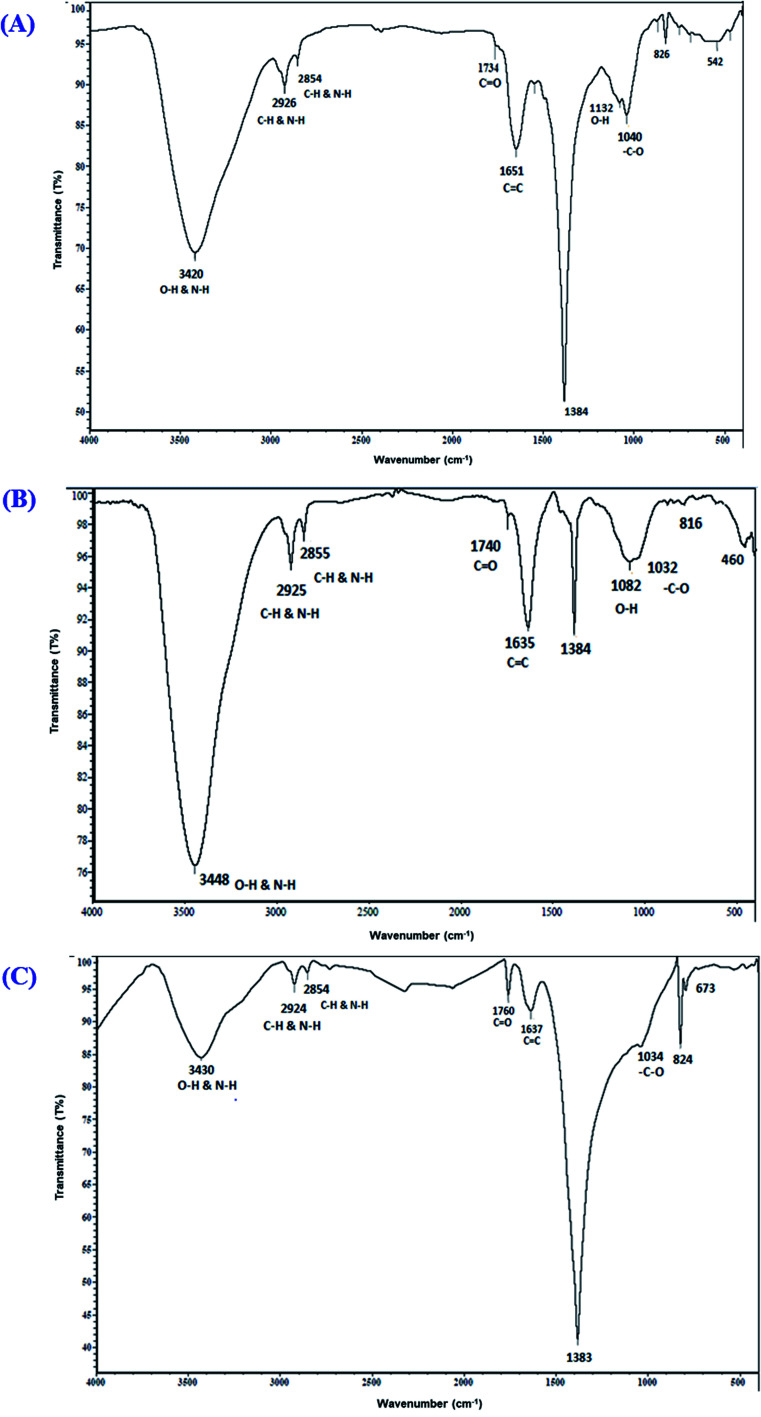
FTIR spectra of the photo-synthesized *O. limnetica* silver nanoparticles (O-AgNPs) recorded as a function of light intensity: (A) 75.90 (fluorescent light), (B) 1276.51 μmol m^−2^ s^−1^ (sunlight) and (C) in the dark.

FTIR spectroscopic analysis was conducted to describe the biomolecules involved in reducing the Ag^+^ ions and in capping and stabilizing the biofabricated O-AgNPs.^46^ The FTIR spectra of AgNPs photo-fabricated using *O. limnetica* are represented in Fig. 2. The FTIR analysis of the photo-synthesized O-AgNPs at the light intensity of 75.90 μmol m^−2^ s^−1^ (fluorescent light) showed 10 major absorption peaks at 3420, 2926, 2854, 1734, 1651, 1384, 1132, 1040, 826 & 542 cm^−1^ (Fig. 2A), while that of the O-AgNPs synthesized under exposure to sunlight (1276.51 μmol m^−2^ s^−1^) revealed 10 major spectral bands (Fig. 2B) having the subsequent wavenumbers 3448, 2925, 2855, 1740, 1635, 1384, 1082, 1032, 816 & 460 cm^−1^. In the dark condition, the FTIR investigation of the O-AgNP reaction mixture (Fig. 2C) revealed 9 major spectral bands at 3430, 2924, 2854, 1760, 1637, 1383, 1034, 824 & 673 cm^−1^. In the spectra obtained, the characteristic broad sharp bands at around 3420–3448 cm^−1^ corresponded to the strong stretching vibration of the hydroxyl groups (O–H) in phenols and flavonoids, and the N–H stretching vibration in peptides and proteins. These results indicate the role of the biological molecules in the *O. limnetica* extract in the bioreduction of silver ions to AgNPs^47^ and the role of proteins in stabilizing and preventing the agglomeration of AgNPs.^48^ The bands observed at 2924 and 2854 cm^−1^ were possibly due to the C–H stretching vibration of alkanes and the N–H bending vibration.^49^ On the other hand, the absorption peaks at 1734–1760 cm^−1^ corresponded to the aldehydic group (C=O),^50^ while the bands in the range 1635–1651 cm^−1^ could be assigned to amide (N–H) stretching and the C=C stretching of the peptide linkages in proteins, which also indicate the involvement of proteins in the stabilization of AgNPs.^38^ The absorption peak at 1384 cm^−1^ could be attributed to the residual amount of AgNO_3_.^51^ Meanwhile, the absorption bands observed at 1132 and 1082 could be matched to the C–N aliphatic and aromatic amine stretching vibrations. The peaks at 1034–1040 cm^−1^ could be assigned to the absorption of the –C–O bond. The absorption bands observed at 816,673, 542 and 460 cm^−1^ could be attributed to the bending areas of the aliphatic chains. Furthermore, the spectral bands at 1384, 1132 & 1034 cm^−1^ corresponded to either sulfur or phosphorus active groups that accomplish both capping and stabilization of AgNPs.^38^

### Statistical optimization of O-AgNP biofabrication using the response surface methodology (RSM)

Herein, a CCD matrix was applied for optimizing the yield of O-AgNPs in terms of optical intensity. CCD is an array of mathematical and statistical practices used for approximating and optimizing the values of the most effectual variables in O-AgNP biofabrication. It was used also to examine their relationships. Depending on the one-factor-based experimental results, four independent variables were chosen as follows: *X*_1_ (initial pH level), *X*_2_ (AgNO_3_ concentration – mM), *X*_3_ (*O. limnetica* extract concentration – mL) and *X*_4_ (incubation period – h) (Table 2). The resultant CCD matrix and the responses of the design are demonstrated in Table 3, which shows thirty-one trails including 16 cube, 8 axial and 7 central points. The seven central points obtained are represented in standard order 25, 26, 27, 28, 29, 30 and 31. The optimum variable levels for the maximum O-AgNP yield (0.8695 nm) were attained in trial number 24, in which *X*_1_ = 6.7 pH, *X*_2_ = 0.3 mM, *X*_3_ = 3.50 mL and *X*_4_ = 48 hours. Optimizing the parameters caused a significant increase in O-AgNP yield, which was 22.45% prior to optimization. The level of significance was measured at a 90% level of confidence, and *P*-values <0.1 were deliberated as important in indicating the pattern of the reciprocal interactions between the variables. The linear coefficient terms (*X*_1_, *X*_2_, *X*_3_, *X*_4_) and quadratic coefficient terms (*X*_1_^2^, *X*_2_^2^, *X*_3_^2^, *X*_4_^2^) demonstrated significant positive responses, whereas the interaction coefficient terms between *X*_1_*X*_2_, *X*_1_*X*_3_, *X*_1_*X*_4_, *X*_2_*X*_3_, and *X*_2_*X*_4_ showed non-significance except for *X*_3_*X*_4_. Upon analyzing the results using multi-way ANOVA (Table 4), the *F*-value (18.96) indicated that the model was significant. The results illustrated that the most significant variables affecting the yield of O-AgNPs were initial pH level, AgNO_3_ concentration (mM), *O. limnetica* extract concentration (mL) and incubation period (h).

**Table 2:** Coded and actual values of the experimental variables used for the CCD matrix

Variable	Variable code	Levels				
		−2	−1	0	1	2
Initial pH level	*X*_1_	4.7	5.7	6.7	7.7	8.7
AgNO_3_ concentration (mM)	*X*_2_	0.1	0.2	0.3	0.4	0.5
*O. limnetica* extract concentration (mL)	*X*_3_	1	2.25	3.50	4.75	6
Incubation period (h)	*X*_4_	48	37.5	27	16.5	6

**Table 3:** Central composite design (CCD) matrix of the four parameters with the experimental values from photo-induced O-AgNP biosynthesis (light intensity: 75.90 μmol m^−2^ s^−1^)

Std. order	Initial pH level (*X*_1_)	AgNO_3_ concentration (mM) (*X*_2_)	*O. limnetica* extract concentration (mL) (*X*_3_)	Incubation period (h) (*X*_4_)	Intensity (nm)
1	5.7	0.2	2.25	16.5	−0.0016
2	7.7	0.2	2.25	16.5	0.0060
3	5.7	0.4	2.25	16.5	−0.0022
4	7.7	0.4	2.25	16.5	0.0391
5	5.7	0.2	4.75	16.5	−0.0221
6	7.7	0.2	4.75	16.5	0.0043
7	5.7	0.4	4.75	16.5	−0.0516
8	7.7	0.4	4.75	16.5	0.3450
9	5.7	0.2	2.25	37.5	0.0326
10	7.7	0.2	2.25	37.5	0.3054
11	5.7	0.4	2.25	37.5	0.2855
12	7.7	0.4	2.25	37.5	0.3064
13	5.7	0.2	4.75	37.5	0.3220
14	7.7	0.2	4.75	37.5	0.4584
15	5.7	0.4	4.75	37.5	0.3866
16	7.7	0.4	4.75	37.5	0.6753
17	4.7	0.3	3.50	27.0	−0.0029
18	8.7	0.3	3.50	27.0	0.1670
19	6.7	0.1	3.50	27.0	0.0427
20	6.7	0.5	3.50	27.0	0.1479
21	6.7	0.3	1.00	27.0	0.1133
22	6.7	0.3	6.00	27.0	0.2255
23	6.7	0.3	3.50	6.0	0.2979
24	6.7	0.3	3.50	48.0	0.8695
25	6.7	0.3	3.50	27.0	0.4324
26	6.7	0.3	3.50	27.0	0.4324
27	6.7	0.3	3.50	27.0	0.4309
28	6.7	0.3	3.50	27.0	0.4319
29	6.7	0.3	3.50	27.0	0.4331
30	6.7	0.3	3.50	27.0	0.4339
31	6.7	0.3	3.50	27.0	0.4324

**Table 4:** Regression statistics of CCD: the regression coefficients and analysis of variance (ANOVA) of the CCD model for the photo-induced O-AgNP biofabrication process using *O. limnetica* extract. df: degree of freedom, *significant values, F: Fishers's function, *P*: level of significance, C. V: coefficient of variation

Source	Sum of squares	df	*F*-value	*P*-value prob > *F*	Coefficient estimate
Model	1.44221	14	18.96	<0.000*	0.4324
*X*_1_	0.09760	1	17.96	0.001*	0.0637
*X*_2_	0.04946	1	9.10	0.008*	0.0454
*X*_3_	0.07833	1	14.41	0.002*	0.0571
*X*_4_	0.53955	1	99.29	0.000*	0.1499
*X*_1_ *X*_2_	0.00579	1	1.06	0.317	0.0190
*X*_1_ *X*_3_	0.01597	1	2.94	0.106	0.0315
*X*_1_ *X*_4_	0.00381	1	0.70	0.415	0.0154
*X*_2_ *X*_3_	0.00586	1	1.08	0.314	0.0191
*X*_2_ *X*_4_	0.00230	1	0.42	0.525	0.0119
*X*_3_ *X*_4_	0.02874	1	5.29	0.035*	0.0423
*X*_1_^2^	0.20075	1	46.68	<0.000*	−0.0941
*X*_2_^2^	0.22055	1	43.45	<0.000*	−0.0908
*X*_3_^2^	0.16561	1	27.54	<0.000*	−0.0723
*X*_4_^2^	0.02788	1	5.13	0.038*	0.0312
Residual	0.08695	16			
Lack of fit	0.08694	10			
Pure error	0.00001	6			
CorTotal	1.52916	30			
PRESS	0.500794				
		*R*-squared			
		Adj *R*-squared			
		Pre *R*-squared			

The second-order regression equation represented the dependence of high AgNP yield on *X*_1_ (initial pH level), *X*_2_ (AgNO_3_ concentration), *X*_3_ (*O. limnetica* extract concentration) and *X*_4_ (incubation period) during the reaction. The interaction between the variables was modeled using a second-order polynomial relation in terms of the coded aspects:

4 *Y* = 0.4324 + 0.0637*X*_1_ + 0.0454*X*_2_ +0.0571*X*_3_ + 0.1499*X*_4_ − 0.0941*X*_1_^2^ − 0.0908*X*_2_^2^ − 0.0723*X*_3_^2^ + 0.0312*X*_4_^2^ + 0.0190*X*_1_*X*_2_ + 0.0315*X*_1_*X*_3_ + 0.0154*X*_1_*X*_4_ + 0.0191*X*_2_*X*_3_ + 0.0119*X*_2_*X*_4_ + 0.0423*X*_3_*X*_4_

where *Y* is the predicted response (silver nanoparticle biofabrication), *X*_1_ is the initial pH, *X*_2_ is the AgNO_3_ concentration, *X*_3_ is the *O. limnetica* extract concentration and *X*_4_ is the incubation period.

**Fig. 3:**
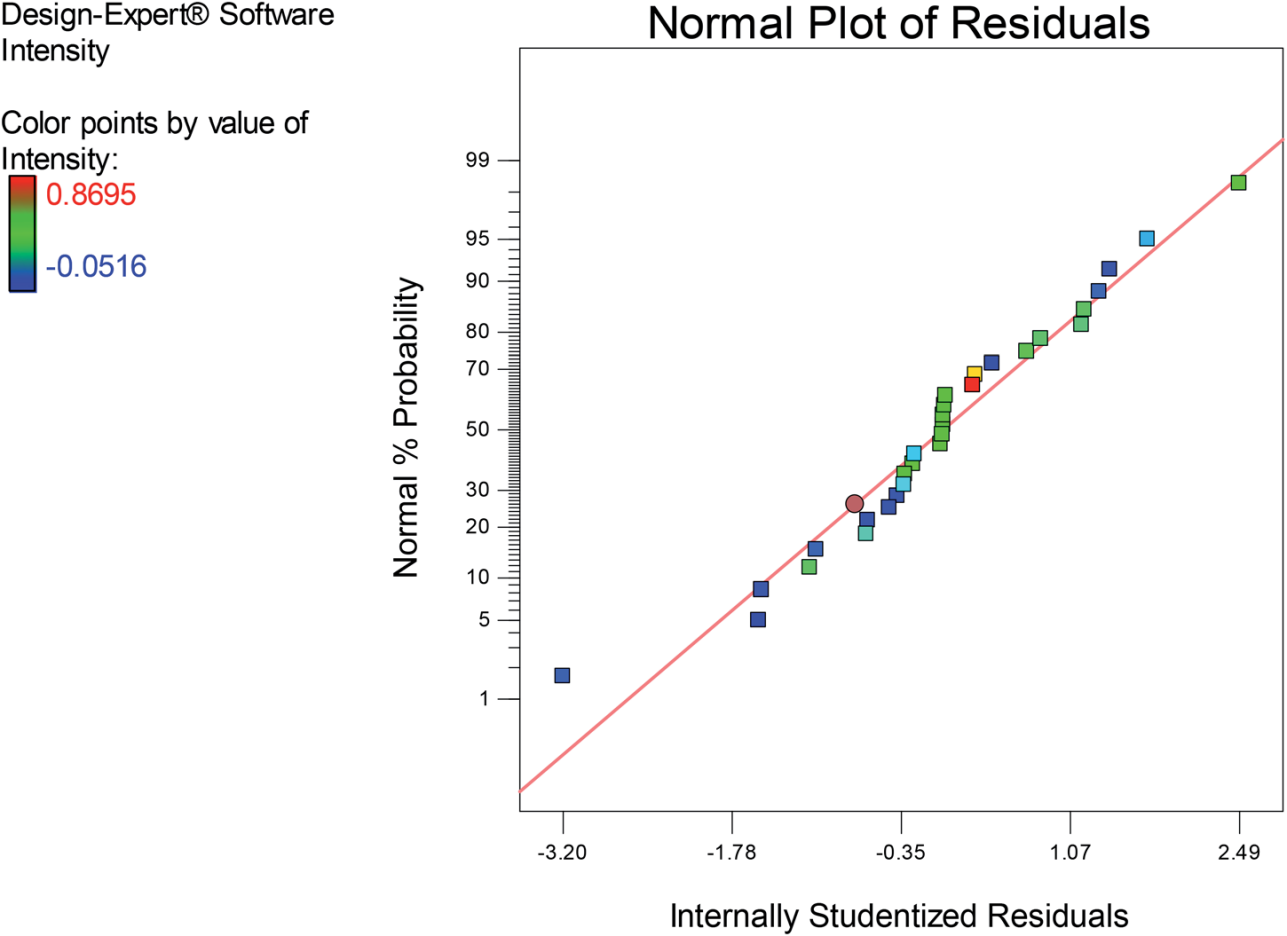
Normal plot of the residuals for O-AgNP biosynthesis based on the second-order polynomial equation.

Subsequently, the coefficient of determination *R*^2^ is used for testing the fitness of the model, in which an *R*^2^ magnitude close to 1 exhibits a good correlation between the experimental and predicted responses.^52^ Our results demonstrated an *R*^2^ value of 94.31, which is in accordance with the adjusted computed coefficient (*R*_Adj_^2^) 89.34, thus referring to the high significance of the model. The normal plot of residuals (Fig. 3) illustrated the fitness of the model as the residuals were normally distributed on the sides of the straight line of the O-AgNPs biosynthesis, demonstrating its validation.

**Fig. 4:**
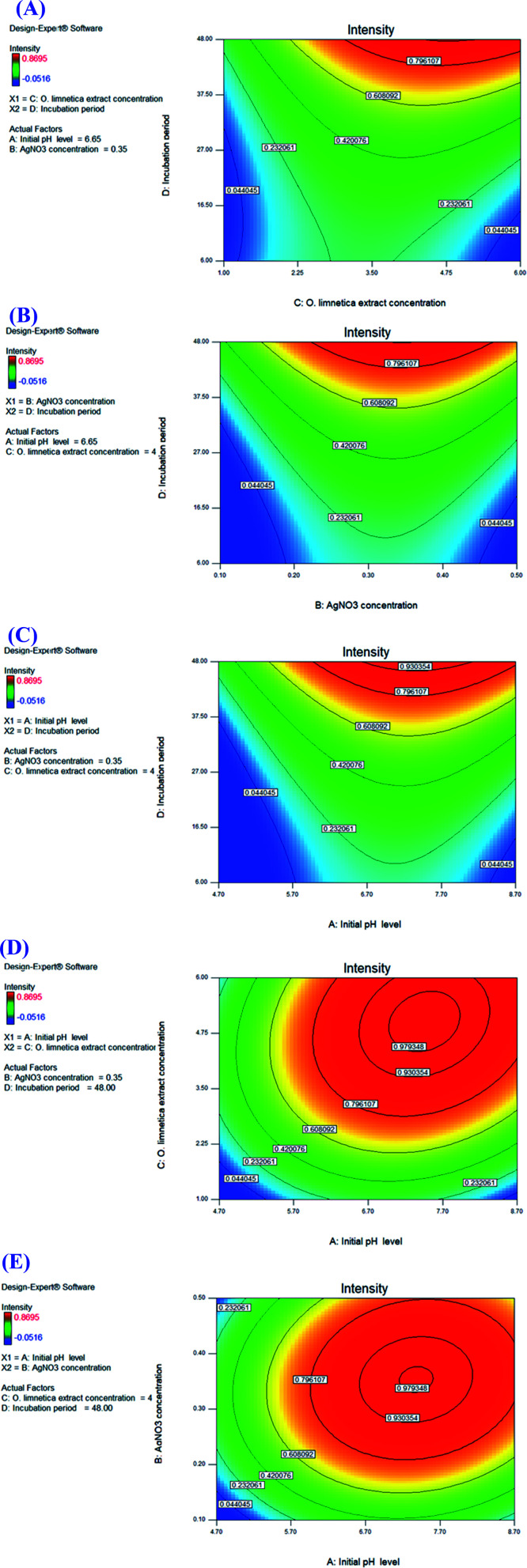
Color contour plots (A–E) showing the interactions between the tested variables for the maximum yield of photo-synthesized O-AgNPs.

The contour plots (Fig. 4) illustrated the interaction between the variables studied for optimizing O-AgNP biosynthesis. Red designates the direction of the optimum condition for each of the suggested responses.

Regarding the interaction between the *O. limnetica* extract concentration and the incubation period (Fig. 4A), the optimum extract concentration range for the intensity of the resultant O-AgNP preparation *i.e.*, response value (*λ* = 430 nm) was 3.36–6 mL, whereas the optimum incubation period was 43.6–48 h. With respect to the interaction between AgNO_3_ concentration and incubation period (Fig. 4B), the optimum AgNO_3_ concentration range for the concerned response (*λ*_430nm_) was (0.22–0.50 mM) since the optimum incubation period was between (42.8–48 h). As for the interaction between the initial pH level and incubation period (Fig. 4C), the optimum initial pH level range for the concerned response (*λ* = 430 nm) was between 5.95 and 8.70 though the optimum incubation period lied between 41.9 and 48 h. For the interaction between the initial pH level and *O. limnetica* extract concentration (Fig. 4D), the observed optimum initial pH level range for the calculated response (*λ* = 430 nm) was 5.89–8.70, while the optimum *O. limnetica* extract concentration ranged from 2.98 to 6 mL. In terms of the relationship between the initial pH level and AgNO_3_ concentration (Fig. 4E), it was observed that the optimum initial pH level range for the concerned response (*λ*_430nm_) was 5.95–8.70, whereas the optimum AgNO_3_ concentration ranged from 0.28 to 0.50 mM.

**Fig. 5:**
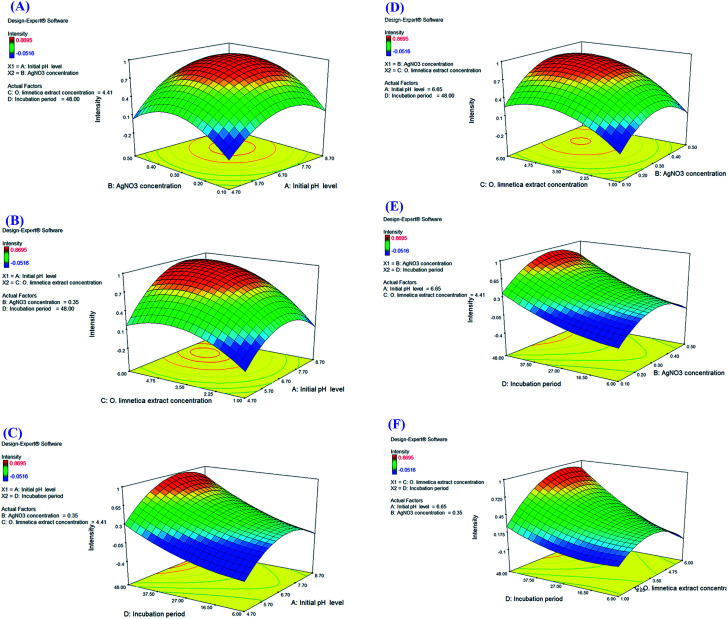
Three-dimensional surface plots (A–F) of the photo-synthesized O-AgNP yield showing the effect of interactions between the independent variables: initial pH level, AgNO_3_ concentration, *O. limnetica* extract concentration and incubation period.

For interpreting the three-dimensional graphs, the regression model was illustrated to indicate the influences of the four investigated variables as well as the mutual possessions of each independent unpredictable variable on O-AgNP biofabrication in terms of peak intensity at 430 nm. The data obtained for the pair-wise interactions of the 4 variables in the silver nanoparticle biosynthesis were illustrated on the *z*-axis against the two independent variables, whereas the other two variables were at zero levels. Fig. 5A signifies the effect of *X*_1_ (initial pH level) and *X*_2_ (AgNO_3_ concentration – mM), while *X*_3_ (*O. limnetica* extract concentration – 4.41 mL) and *X*_4_ (incubation period – 48 h) were detained at their zero points. The maximum silver nanoparticle yield was attained with the initial pH at 6.7, whereas the production increased rapidly with decreasing acidity accompanied by an increase in AgNO_3_ concentration until the middle point. Shifting the pH towards the alkaline side (pH 7.7 and pH 8.7) induced a gradual decrease in O-AgNP formation (0.7 nm and 0.2 nm), respectively. According to previous work,^13^ pH affects the electric charges of biomolecules, as well as the capping agents, inducing variations in their capability to bind and generate zero-charge metal ions. Fig. 5B illustrates the response for *X*_1_ (initial pH level) and *X*_3_ (*O. limnetica* extract concentration), while *X*_2_ (AgNO_3_ concentration = 0.35 mM) and *X*_4_ (incubation period = 48 h) were set at their zero levels. The highest silver nanoparticle production was achieved at pH 6.7. The silver nanoparticle biosynthesis improved gradually on rising the *O. limnetica* extract concentration.^22^ The interaction of *X*_1_ (initial pH level) and *X*_4_ (incubation period) during O-AgNP biosynthesis is demonstrated in Fig. 5C, where *X*_2_ (AgNO_3_ concentration = 0.35 mM) and *X*_3_ (*O. limnetica* extract concentration = 4.41 mL) were kept at their zero levels. The maximum O-AgNPs yield was designated at pH 6.7. A marked increase in O-AgNP biosynthesis was noticed with an increasing incubation period. This is in agreement with the discovery of Roychoudhury *et al.*,^53^ who synthesized AgNPs using *Lyngbya majuscula* extract after 72 h, whereas the time required while using a cyanobacterial extract (*Synechococcus* sp.) ranged between 24 h to 72 h.^54^ The effect of *X*_2_ (AgNO_3_ concentration) and *X*_3_ (*O. limnetica* extract concentration) on O-AgNP biosynthesis is presented in Fig. 5D, whereas *X*_1_ (initial pH level = 6.65) and *X*_4_ (incubation period = 48 h) are set at their zero levels. Besides, there a distinct upsurge in O-AgNP biosynthesis was noticed on improving the AgNO_3_ concentration and *O. limnetica* extract concentration. The interaction between *X*_2_ (AgNO_3_ concentration) and *X*_4_ (incubation period) is illustrated in Fig. 5E, whereas *X*_1_ (initial pH level = 6.65) and *X*_3_ (*O. limnetica* extract concentration = 4.41 mL) were held at their zero points. The maximum O-AgNPs yield was detected near the central level of both the incubation period and AgNO_3_ concentration. The interaction between *X*_3_ (*O. limnetica* extract concentration) and *X*_4_ (incubation period) is demonstrated in Fig. 5F, whereas *X*_1_ (initial pH level = 6.65) and *X*_2_ (AgNO_3_ concentration = 0.35 mM) were set at their zero levels. Fig. 5F illustrates that the maximum O-AgNPs yield was detected on increasing the *O. limnetica* extract concentration and incubation period.

### Model validation

On applying mathematical models in addition to the three-dimensional interaction-surface response illustrations, the best settings for every individual response variable (*i.e.* single-factor optimization) that allowed the maximum biosynthesis of O-AgNPs yielded 0.87 nm optical intensity against the predicated value. This illustrated a high level of model precision, which was confirmed by model validation under experimental conditions. Finally, the data revealed that the optimized values of the variables for silver nanoparticle biosynthesis were as follows: initial pH level (6.7), AgNO_3_ concentration (0.3 mM), *O. limnetica* extract concentration (3.50 mL) and incubation period (48 h), which were fixed for further experiments.

### Microstructural analysis of the O-AgNPs

**Fig. 6:**
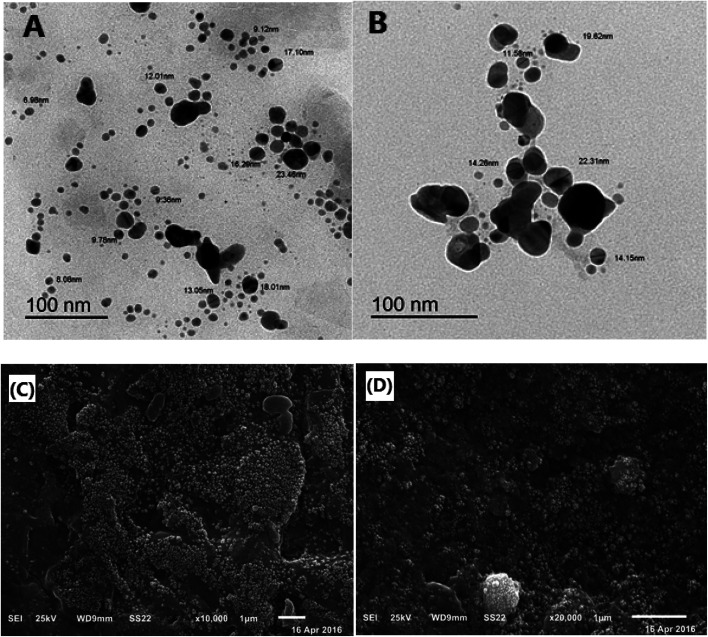
Microstructural analysis of the optimized O-AgNPs: (A) & (B) transmission electron (C) & (D) scanning electron micrographs at the light intensities 75.90 and 1276.51 μmol m^−2^ s^−1^ (sunlight), respectively.

The morphological characteristics and the microstructure of O-AgNPs photo-synthesized at the optimum conditions (48 h at light intensity 75.90 μmol m^−2^ s^−1^, 0.3 mM AgNO_3_, 3.50 mL *O. limnetica* and pH 6.7) and (30 min sunlight (1276.51 μmol m^−2^ s^−1^), 0.5 mM AgNO_3_, 5 mL *O. limnetica* solution pH 6.7) were detected by SEM and TEM. First, the TEM images (Fig. 6A and B) demonstrated the shape and size of the biosynthesized O-AgNPs. They exposed that the O-AgNPs had a quasi-spherical shape and anisotropic nanostructures, and the size range was between 6.98–23.48 nm in the case of light-induced synthesis (75.90 μmol m^−2^ s^−1^) and 11.58–22.31 nm in that using sunlight (1276.51 μmol m^−2^ s^−1^). Moreover, the particles were well-dispersed without agglomeration.^55^ Furthermore, Fig. 6C and D show the SEM images of O-AgNPs, which clearly elucidate the presence of smaller and monodispersed AgNPs.

### Particle size and zeta potential analysis of the O-AgNPs

**Fig. 7:**
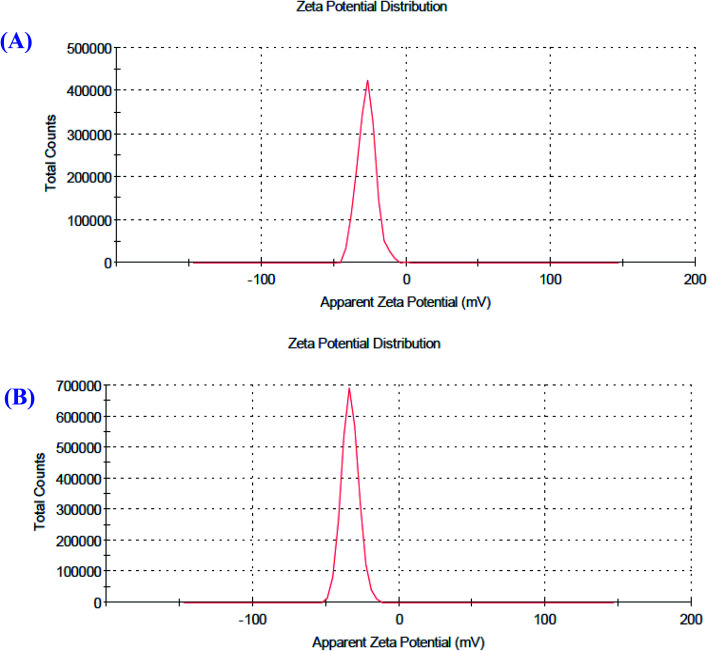
Zeta potential distribution profile of the optimized photo-synthesized O-AgNPs (A) at light intensity 75.90 and (B) at 1276.51 μmol m^−2^ s^−1^ (sunlight).

**Fig. 8:**
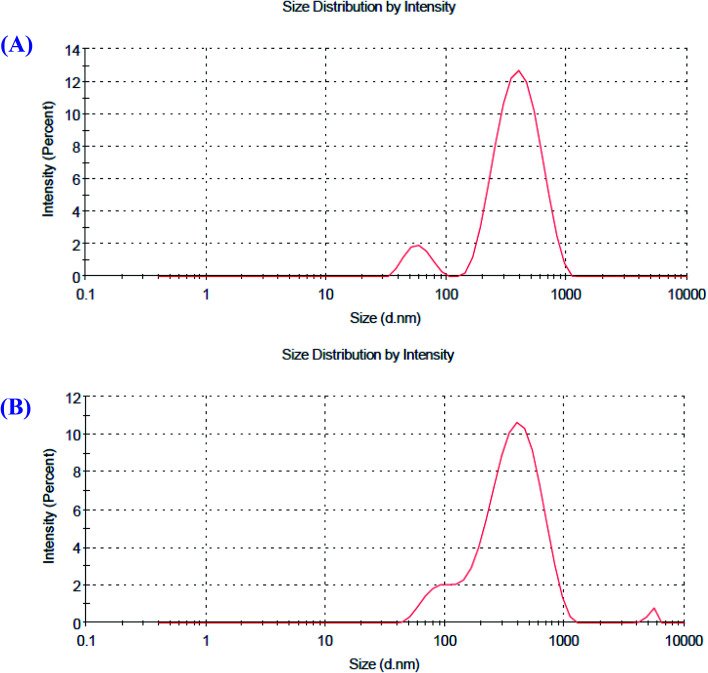
Particle size distribution of the optimized photo-synthesized O-AgNPs (A) at light intensity 75.90 and (B) at 1276.51 μmol m^−2^ s^−1^ (sunlight).

Zeta potential is a factor that reveals the surface charges and stability of the prepared NPs. In addition, charges have a significant influence on the nanoparticle distribution and cellular uptake. Charges explain the robust resistive forces amongst particles that avoid the aggregation and stabilize the NPs in the medium. The zeta potential values of O-AgNPs photo-synthesized at the optimum conditions (48 h at light intensity 75.90 μmol m^−2^ s^−1^) and (30 min sunlight 1276.51 μmol m^−2^ s^−1^) were −27.4 and −33.4 mV with the standard deviations of 6.38 and 5.86 mV and conductivity values of 2.24 and 2.32 mS cm^−1^, respectively (Fig. 7A and B), indicating negatively charged surfaces in the produced nanoparticles. These results are in agreement with the zeta potential of AgNPs (−31.8 mV) biosynthesized using phycocyanin extracted from *Nostoc linckia*.^56^ Moreover, El-Naggar *et al.*^57^ documented that the higher electronegativity of the phycoerythrin-mediated AgNPs, characterized by the zeta potential value equal to −32.0 mV, may be explained by the presence of effective functional constituents that act as capping agents in the phycobiliprotein extract. The zeta potential results demonstrated that O-AgNPs were stable because of electrostatic repulsion, which can be enhanced by altering the physical or chemical capping agent. Moreover, this good stability may be due to the occurrence of bio-organic constituents that act as capping and reducing agents. Additionally, this is very vital as the nanoparticles are intended for therapeutic purposes. According to Fig. 8A and B, the DLS analysis was performed for detecting the hydrodynamic diameters of the biofabricated O-AgNPs, which were found to have average diameters of 266.2 and 272.9 at the optimum conditions (48 h at light intensity 75.90 μmol m^−2^ s^−1^) and (30 min sunlight 1276.51 μmol m^−2^ s^−1^) and the polydispersity indices (PdI) 0.451 and 0.399, respectively, which revealed that the O-AgNPs were of monocular nature.^58^ The PdI measurement is extremely vital as it represents the nanoparticle distribution and is also used as a sign of the uniformity of size distribution.^59^ Salvia-Trujillo *et al.*^60^ reported that a PdI value close to 1 theoretically indicates heterogeneous size distribution of the particles, whereas a PdI of 0 denotes monocular populations. The average size value of O-AgNPs varied between TEM and DLS analysis, which might be assigned to the agglomeration of particles due to the heterogeneous aggregated dispersion behavior.

### Antioxidant capacity of O-AgNPs

#### DPPH assay

**Fig. 9:**
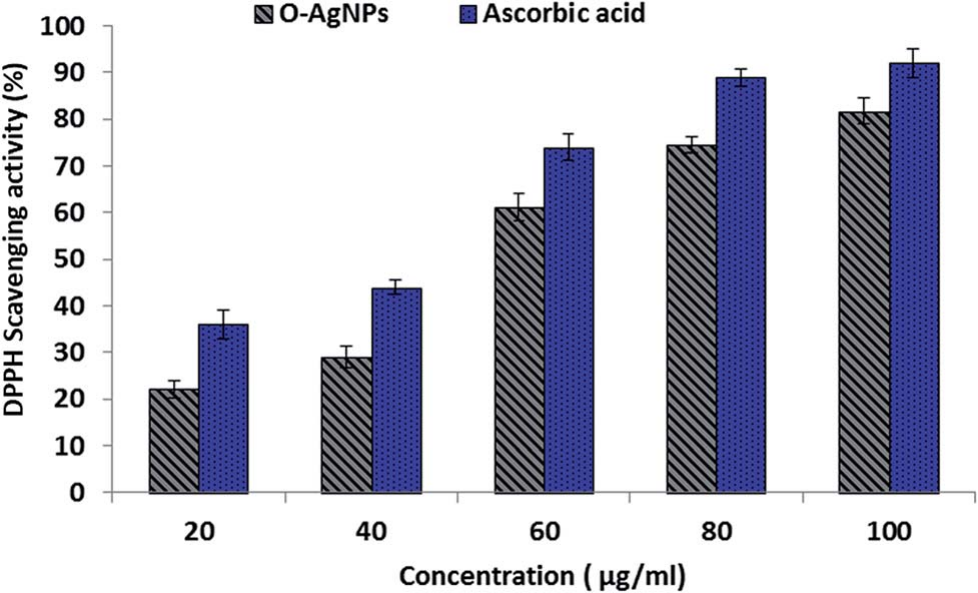
DPPH free-radical-scavenging activity of the optimally photo-synthesized O-AgNPs (48 h at light intensity 75.90 μmol m^−2^ s^−1^, 0.3 mM AgNO_3_, 3.50 mL *O. limnetica* and pH 6.7).

The DPPH molecule that owns a nitrogen free-radical is susceptible to destruction by a free radical scavenger, and this is expended to study the power of oxidative compounds to function as proton radical scavengers or hydrogen donors.^61^ O-AgNPs are an abundant source of hydrogen as they are protected by capping agents, such as proteins, free amino acids, as well as sulfur-containing amino acid derivatives.^22^ The violet free-radical (DPPH) solution turned yellow after the alteration of diphenypicrylhydrazyl (free radical) to diphenylpicryhydrazine (non-free radical) due to the removal of hydrogen atoms that were bound to the O-AgNPs surface.^62^ Consequently, the level of color reduction was applied to evaluate the extent of the free-radical-scavenging activity of O-AgNPs. This scavenging assay revealed the effectual inhibition potentiality of O-AgNPs relative to ascorbic acid, which was used as the standard (Fig. 9). Data illustrated that the DPPH-scavenging potentiality of O-AgNPs progressively increased in a dose-dependent way since the maximum inhibition action was recorded at 100 μg mL^−1^ O-AgNPs, which provided 81.74% DPPH-scavenging activity.^63^ Additionally, the free-radical-scavenging activity rose, and the lesser IC_50_ value reflected better protective action. The IC_50_ value of the photo-synthesized O-AgNPs was 53.2 μg mL^−1^. This result indicated that the photo-synthesized O-AgNPs displayed strong antioxidant potential (DPPH-scavenging activity).

### ABTS^+^ radical-scavenging (antioxidant) activity

**Fig. 10:**
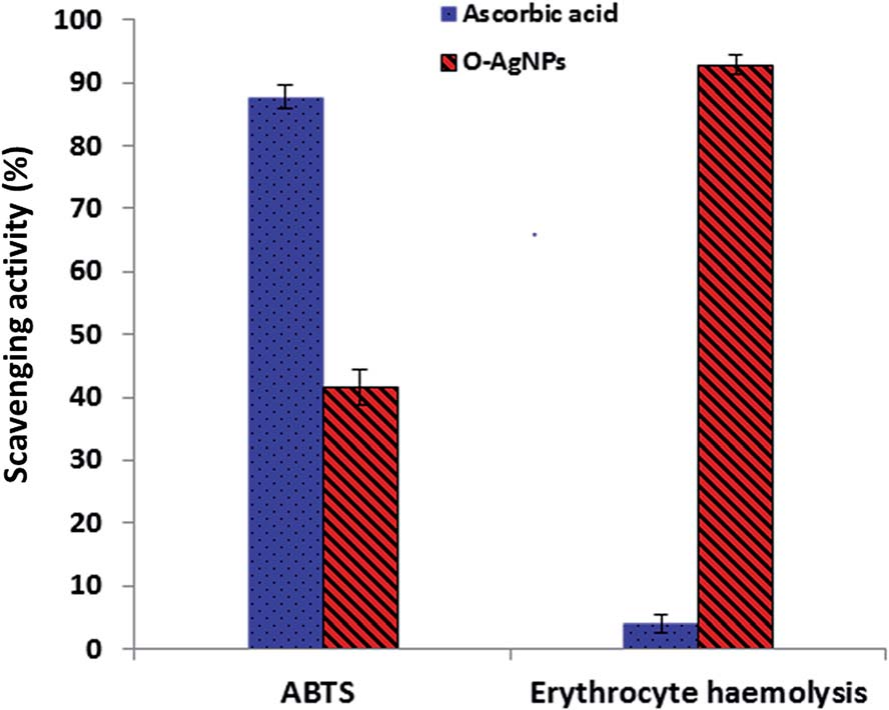
Antioxidant activity screening assay of the optimally photo-synthesized O-AgNPs: ABTS assay and erythrocyte haemolysis.

The ABTS assay depends on electron transfer and can detect various types of antioxidant substances.^64^ O-AgNPs (80 μg mL^−1^) demonstrated an antioxidant activity of 41.7% inhibition of the radical compared with the standard antioxidant ascorbic acid, which showed the activity of 87.8% inhibition (Fig. 10). The ABTS radical cation interacted with O-AgNPs and the hydrogen atoms were transferred to ABTS^+^, causing neutralization and in turn, showing its efficient scavenging activity.

### Anti-hemolytic assay

Erythrocytes are oversensitive to oxidative damage due to the chemical composition of the erythrocyte membrane, which is characterized by a high content of polyunsaturated fatty acids and the presence of high oxygen concentration in addition to hemoglobin, which are effective organizers of oxidative procedures.^65^ When a free radical generates lipid peroxyl radicals, the lipid membrane can be attacked and altered to lipid hydroperoxides, resulting in destructive effects on membrane biology.^65^ The solvable hydrophilic azo-compound AAPH produces 2-amidinopropyl radicals, also-called C-radicals, *via* a temperature-based metabolic pathway. In the presence of oxygen, the formed C-radicals produce peroxyl radicals, resulting in increased hemolysis based on the time of erythrocyte incubation with AAPH.^66^ The preparation of O-AgNPs with erythrocytes, when subjected to the AAPH free radicals, demonstrated inhibition of erythrocyte hemolysis (761%) *i.e.*, O-AgNPs revealed anti-hemolytic potential (92.93% inhibition) (Fig. 10). Here, hemolysis was reduced in comparison with the complete erythrocyte hemolysis attained when the erythrocytes and AAPH were incubated without O-AgNPs. Therefore, treatment with AgNPs protects erythrocytes from oxidative damage. Ascorbic acid demonstrated 4.0% erythrocyte hemolysis.^66^

## Conclusion

This study demonstrates a photo-induced biological method towards the green preparation of stable and quasi-spherical AgNPs using the *O. limnetica* aqueous extract. The central composite design (CCD) was applied to optimize the factors affecting O-AgNP biosynthesis and accomplish the maximum production of O-AgNPs (manufactured at light intensity 75.90 μmol m^−2^ s^−1^). High-productivity O-AgNP biofabrication could be attained through a facile, rapid, cost-effective, eco-friendly protocol. The optimum condition for the synthesis process under exposure to sunlight was detected through the one-factor-at-a-time approach, based on which 0.5 mM AgNPs concentration, 5 mL *O. limnetica* solution, pH 6.7 and 30 min sunlight (1276.51 μmol m^−2^ s^−1^) were determined as the optimal values. Physicochemical characterization using UV-Vis spectroscopy, FTIR, TEM, SEM and particle size and zeta potential ascertained the synthesis of O-AgNPs. The zeta potential values of the optimized photo-induced O-AgNPs were −27.4 and −33.4 mV with the standard deviations of 6.38 and 5.86 mV and conductivity values of 2.24 and 2.32 mS cm^−1^ nm in the case of light-induced (75.90 μmol m^−2^ s^−1^) and sunlight-induced syntheses (1276.51 μmol m^−2^ s^−1^), respectively, indicating good stability of the produced nanoparticles. The *in vitro* antioxidant assays revealed that the biosynthesized O-AgNPs exhibited great radical-scavenging potential in terms of DPPH and ABTS, and erythrocyte haemolysis assays. These results indicate that O-AgNPs can be beneficial as free radical inhibitors and therapeutic agents for treating free-radical-related pathological damage.

## Conflicts of interest

The authors declare no competing of interests.

## Supplementary Material
