# Expression of Concern: Statistical optimization of photo-induced biofabrication of silver nanoparticles using the cell extract of *Oscillatoria limnetica*: insight on characterization and antioxidant potentiality

**DOI:** 10.1039/d3ra90110f

**Published:** 2023-11-08

**Authors:** Rasha A. Abo-Elmagd, Mervat H. Hussein, Ragaa A. Hamouda, Ahmed Esmail Shalan, Ahmed Abdelrazak

**Affiliations:** a Botany Department, Faculty of Science, Mansoura University Mansoura Egypt ahmed_bt@mans.edu.eg; b Department of Biology, Faculty of Sciences and Arts Khulais, University of Jeddah Jeddah Saudi Arabia ragaahom@yahoo.com; c Department of Microbial Biotechnology, Genetic Engineering & Biotechnology Research Institute, Sadat University Sadat City Egypt; d Central Metallurgical Research and Development Institute (CMRDI) P.O. Box 87, Helwan Cairo 11421 Egypt a.shalan133@gmail.com; e BCMaterials, Basque Center for Materials, Applications and Nanostructures Martina Casiano, UPV/EHU Science Park, Barrio Sarriena s/n Leioa 48940 Spain

## Abstract

Expression of Concern for ‘Statistical optimization of photo-induced biofabrication of silver nanoparticles using the cell extract of *Oscillatoria limnetica*: insight on characterization and antioxidant potentiality’ by Rasha A. Abo-Elmagd *et al.*, *RSC Adv*., 2020, **10**, 44232–44246, DOI: https://doi.org/10.1039/D0RA08206F.

The Royal Society of Chemistry is publishing this expression of concern in order to alert readers that concerns have been raised regarding the reliability of the UV-vis absorption spectra in Fig. 1. An investigation is underway, and an expression of concern will continue to be associated with the article until a final outcome is reached.

Laura Fisher

2nd November 2023

Executive Editor, *RSC Advances*

## Supplementary Material

